# Transcriptomic Pattern of Genes Regulating Protein Response and Status of Mitochondrial Activity Are Related to Oocyte Maturational Competence—A Transcriptomic Study

**DOI:** 10.3390/ijms20092238

**Published:** 2019-05-07

**Authors:** Błażej Chermuła, Maciej Brązert, Michal Jeseta, Katarzyna Ożegowska, Ievgenia Kocherova, Maurycy Jankowski, Piotr Celichowski, Patrycja Sujka-Kordowska, Aneta Konwerska, Hanna Piotrowska-Kempisty, Joanna Budna-Tukan, Paweł Antosik, Dorota Bukowska, Marie Machatkova, Klaus P. Brussow, Mariusz T. Skowroński, Leszek Pawelczyk, Małgorzata Bruska, Michał Nowicki, Bartosz Kempisty

**Affiliations:** 1 Division of Infertility and Reproductive Endocrinology, Department of Gynecology, Obstetrics and Gynecological Oncology, Poznan University of Medical Sciences, 60-535 Poznań, Poland; blazej.chermula@wp.pl (B.C.); maciejbrazert@ump.edu.pl (M.B.); katarzyna.ozegowska@ump.edu.pl (K.O.); pawelczyk.leszek@ump.edu.pl (L.P.); 2 Department of Obstetrics and Gynecology, University Hospital and Masaryk University, 601 77 Brno, Czech Republic; jeseta@gmail.com; 3 Department of Anatomy, Poznan University of Medical Sciences, 60-781 Poznań, Poland; kocherova.evgenia@gmail.com (I.K.); m.jankowski.14@aberdeen.ac.uk (M.J.); mbruska@ump.edu.pl (M.B.); 4 Department of Histology and Embryology, Poznan University of Medical Sciences, 60-781 Poznań, Poland; p.celichowski@gmail.com (P.C.); psujka@ump.edu.pl (P.S.-K.); akonwer@ump.edu.pl (A.K.); joanna.budna@wp.pl (J.B.-T.); mnowicki@ump.edu.pl (M.N.); 5 Department of Toxicology, Poznan University of Medical Sciences, 61-631 Poznań, Poland; hpiotrow@ump.edu.pl; 6 Veterinary Center, Nicolaus Copernicus University in Torun, 87-100 Toruń, Poland; pantosik@umk.pl (P.A.); dbukowska@umk.pl (D.B.); prof.bruessow@gmail.com (K.P.B.); skowron@uwm.edu.pl (M.T.S.); 7 Veterinary Research Institute, 621 00 Brno, Czech Republic; machatkova@vri.cz

**Keywords:** pig, oocyte maturation, microarray, mitochondrial activity

## Abstract

This paper aims to identify and describe new genetic markers involved in the processes of protein expression and modification reflected in the change of mitochondrial activity before and after in vitro maturation of the oocyte. Porcine oocytes collected from the ovaries of slaughtered landrace gilts were subjected to the process of in vitro maturation. Transcriptomic changes in the expression profile of oocyte genes involved in response to hypoxia, the transmembrane protein receptor serine threonine kinase signaling pathway, the “transforming growth factor β receptor signaling pathway”, “response to protein stimulus”, and “response to organic substance” were investigated using microarrays. The expression values of these genes in oocytes was analyzed before (immature) and after (mature) in vitro maturation, with significant differences found. All the significantly altered genes showed downregulation after the maturation process. The most changed genes from these gene ontologies, *FOS*, *ID2*, *VEGFA*, *BTG2*, *CYR61*, *ESR1*, *AR*, *TACR3*, *CCND2*, *CHRDL1*, were chosen to be further validated, described and related to the literature. Additionally, the mitochondrial activity of the analyzed oocytes was measured using specific dyes. We found that the mitochondrial activity was higher before the maturation process. The analysis of these results and the available literature provides a novel insight on the processes that occur during in vitro oocyte maturation. While this knowledge may prove to be useful in further research of the procedures commonly associated with in vitro fertilization procedures, it serves mostly as a basic reference for further proteomic, in vivo, and clinical studies that are necessary to translate it into practical applications.

## 1. Introduction

Oocyte growth and maturation is a very dynamic process taking place in an environment controlled by many factors. Most roles in the process of folliculogenesis and oogenesis are played by hormones. These include mainly FSH, LH (produced by the pituitary gland), and steroid hormones that directly or indirectly affect the cumulus cells (CCs) and granulosa cells (GCs), inducing their growth and differentiation.

In the maturing oocyte, synthesis of many factors occurs, playing a significant role in oocyte development as well as in the formation and activation of somatic cells directly communicating with the oocyte [1,2]. These factors include bone morphogenetic proteins (BMPs) and growth differentiation factor 9 (GDF9), both belonging to the transforming growth factor β (TGF-β) family. Considering the bi-directional communication between the oocyte and the surrounding granulosa cells, the disturbances of these pathways may expose the female gamete to numerous cellular stress factors, causing disorders in the synthesis of cellular proteins as well as mitochondrial activity. The causes of these abnormalities, which are associated with disturbances of signaling pathways and molecular mechanisms involved in oocyte maturation, remain unclear. These anomalies may lead to disturbances during the first and second meiotic division. Hence, the current research focuses on genetic factors affecting the quality of oocytes and their in vivo or in vitro maturation. It is important to discover the factors affecting disruptions of the oocyte maturation process. In our previous reports about gene transcripts in immature oocytes and after their maturation, we attempt to identify genes involved in processes such as proliferation, migration, apoptosis [3]; female sex differentiation [4], or genes involved in the regulation of RNA metabolic processes [5].

It has been proven that one of the main factors determining the oocyte’s developmental ability, which ensures its proper fertilization, is mitochondrial DNA [6]. One of the main functions of the mitochondria is generation of energy in the form of adenosine triphosphate (ATP). This power is necessary for achieving homeostasis and basic functioning of the oocyte and other cells [7]. So far, many studies on various animal species, as well as human oocytes, have been conducted and published [8,9,10]. Mostly, they have focused on the number of mtDNA copies and appearing mutations. Our approach was to investigate the determination of the transcriptomic pattern that may cause different protein expression in porcine in vitro matured oocytes, resulting in a change of mitochondrial activity. In our research, particular attention was paid to the analysis of five ontological groups, which included genes directly or indirectly related to the basic mitochondrial activities.

Microarray assays have recently become one of the most important methods used to analyze the cellular transcriptomic profile. This technique provides the opportunity to learn and identify mRNA transcripts that are involved in a variety of physiological processes of the oocyte. The use of microarrays made it possible to discover new genes or ontological groups directly or indirectly involved in many processes regulating the expression of proteins in various states of cellular stress [11,12,13].

This study attempts to compare oocyte transcriptomic patterns before and after in vitro maturation. Genes involved in the regulation of the synthesis of proteins expressed in hypoxia states and the basic signaling pathways involved in the modification of synthesized proteins during oocyte maturation were analyzed. Particular attention was paid to the expression of the genes involved in protein response and mitochondrial activity before and after in vitro maturation.

## 2. Results

Whole transcriptome profiling using Affymetrix microarrays allowed us to analyze the gene expression changes in freshly isolated oocytes before the in vitro procedure (before IVM), and in relation to after in vitro maturation (after IVM). Using an Affymetrix^®^ Porcine Gene 1.1 ST Array we examined the expression of 12,258 porcine transcripts. Genes with a fold change higher than |2| and with a corrected *p*-value lower than 0.05 were considered as differentially expressed. This set of genes consisted of 419 different transcripts. Subsequently, the genes were used for identification of significantly enriched gene ontology biological process (GO BP) terms.

**Figure 1:**
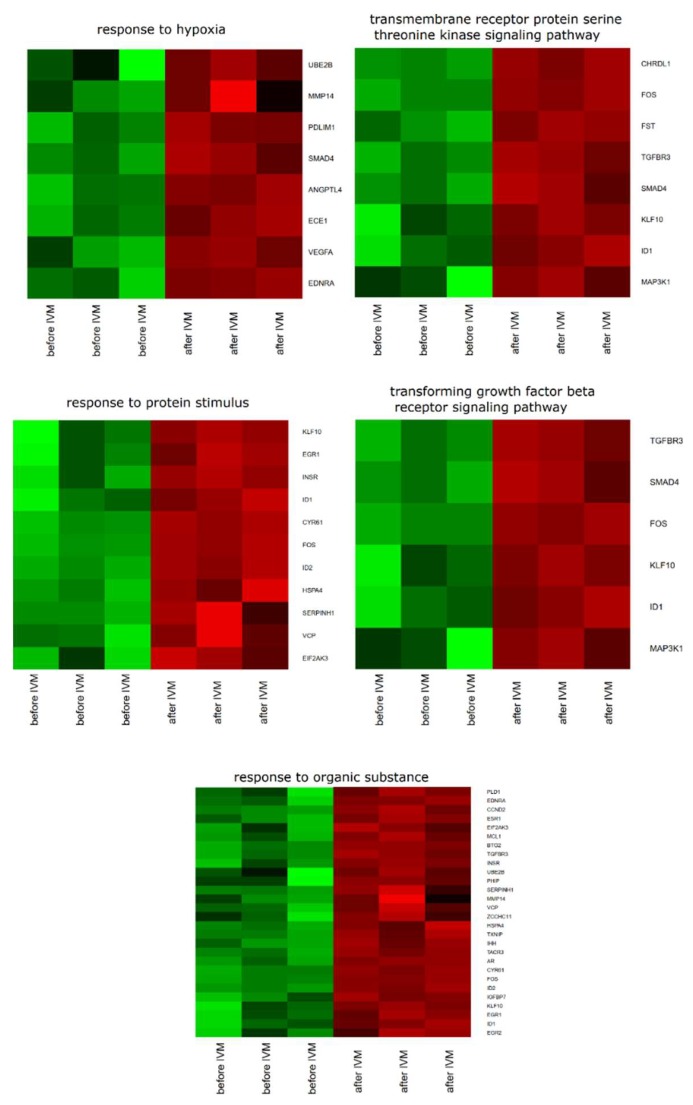
Heat map representations of differentially expressed genes belonging to response to hypoxia, “response to organic substance”, “response to protein stimulus”, the “transforming growth factor β receptor signaling pathway”, and “transmembrane receptor protein serine threonine kinase signaling pathway” gene ontology biological process (GO BP) terms. Arbitrary signal intensity acquired from microarray analysis is represented by colors (green, higher; red, lower expression). Log2 signal intensity values for any single gene were resized to Row z-Score scale (from −2, the lowest expression, to +2, the highest expression for a single gene).

Database for Annotation, Visualization and Integrated Discovery (DAVID) software was used for extraction of the genes belonging to the regulation of response to hypoxia, “response to organic substance”, “response to protein stimulus”, the “transforming growth factor β receptor signaling pathway”, and “transmembrane receptor protein serine threonine kinase signaling pathway” GO BP. We found that 36 genes from these GO BP terms were significantly represented in down-regulated gene sets. This sets of genes were subjected to a hierarchical clusterization procedure and presented as heat maps (Figure 1).

**Figure 2:**
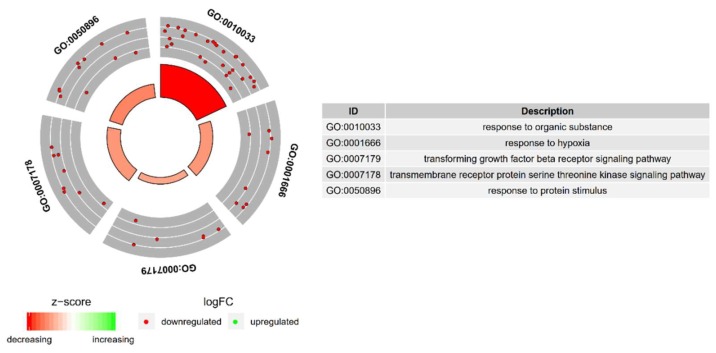
The circle plot showing the differently expressed genes and z-scores of the response to hypoxia, “response to organic substance”, “response to protein stimulus”, the “transforming growth factor β receptor signaling pathway”, and “transmembrane receptor protein serine threonine kinase signaling pathway” GO BP terms. The outer circle shows a scatter plot for each term of the fold change of the assigned genes. Green circles display up-regulation and red ones down-regulation. The inner circle shows the z-score of each GO BP term. The width of each bar corresponds to the number of genes within GO BP term and the color corresponds to the z-score.

A set of the differentially expressed genes belonging to response to hypoxia, “response to organic substance”, “response to protein stimulus”, the “transforming growth factor β receptor signaling pathway”, and “transmembrane receptor protein serine threonine kinase signaling pathway” GO BP terms with their official gene symbols, fold changes in expression, corrected *p*-values and LogFC is shown in Table 1. The enrichment of each GO BP term was calculated as a z-score and shown on the circle diagram (Figure 2).

**Figure 3:**
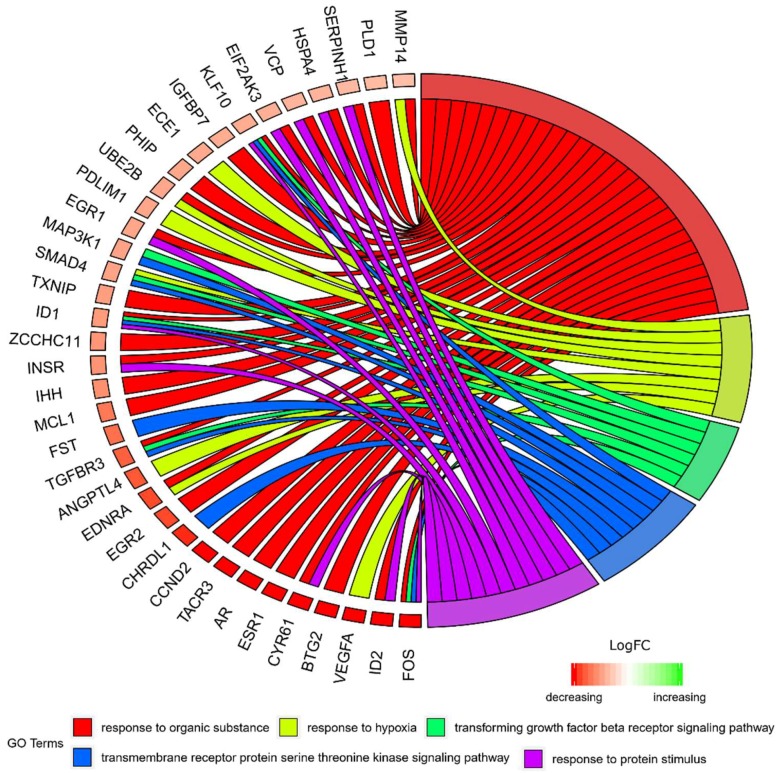
The representation of the mutual relationship between differently expressed genes that belong to the response to hypoxia, “response to organic substance”, “response to protein stimulus”, the “transforming growth factor β receptor signaling pathway”, and “transmembrane receptor protein serine threonine kinase signaling pathway” GO BP terms. The ribbons indicate which gene belongs to which categories. The middle circle represents logarithm from fold change (LogFC). The genes were sorted by logFC from most to least changed gene. The color of the each LogFC bar corresponds with LogFC value.

**Figure 4:**
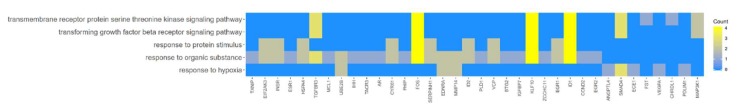
Heatmap showing the gene occurrence between differently expressed genes that belongs to the response to hypoxia, “response to organic substance”, “response to protein stimulus”, the “transforming growth factor β receptor signaling pathway”, and “transmembrane receptor protein serine threonine kinase signaling pathway” GO BP terms. The yellow color is associated with gene occurrence in the GO term. The intensity of the color is corresponding to amount of GO BP terms that each gene belongs to.

Moreover, in gene ontology databases, genes that formed one particular GO group can also belong to other different GO term categories. For this reason, we have explored the gene intersections between selected gene ontology biological process (GO BP) terms. The relation between those GO BP terms is presented as a circle plot (Figure 3) as well as a heatmap (Figure 4). Both Figure 3 and Figure 4 present the membership of the genes in particular ontological group. It needs to be noted that some of the genes are assigned to more than one gene ontology. From the group of genes of the highest expression, *VEGFA, BTG2*, *ESR1, AR*, *TACR3, CCND2*, *CHRDL1* are characteristic for singular GOs.

**Figure 5:**
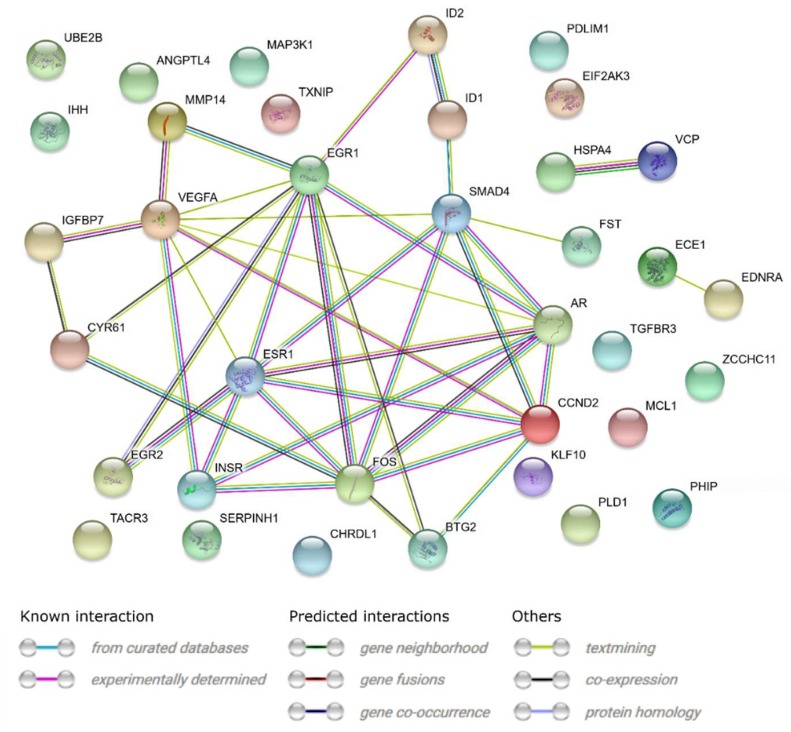
STRING-generated interaction network between genes that belongs to the “response to hypoxia”, “response to organic substance”, “response to protein stimulus”, “transforming growth factor β receptor signaling pathway” and “transmembrane receptor protein serine threonine kinase signaling pathway” GO BP terms. The intensity of the edges reflects the strength of interaction score.

**Figure 6:**
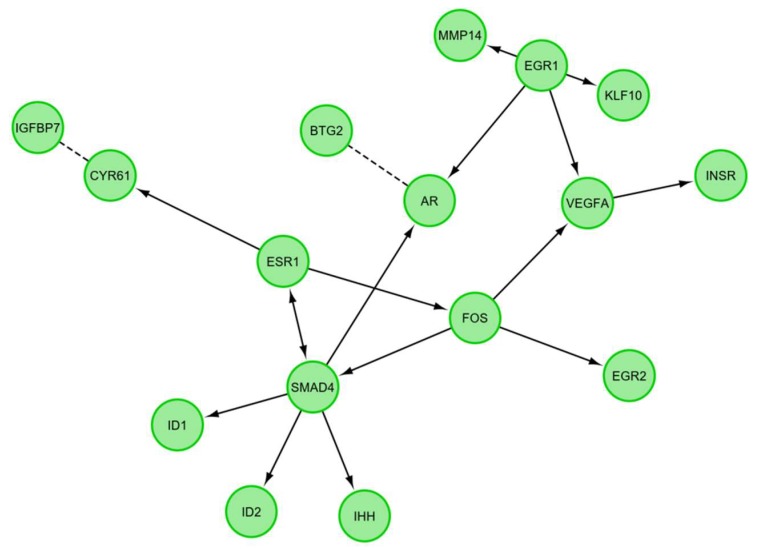
Functional interaction (FI) between differently expressed genes that belongs to the “response to hypoxia”, “response to organic substance”, “response to protein stimulus”, the “transforming growth factor β receptor signaling pathway”, and “transmembrane receptor protein serine threonine kinase signaling pathway”. In following figure “→“ stands for activating/catalyzing for FIs extracted from complexes or inputs, and “---” for predicted FIs.

A STRING-generated interaction network was created for differentially expressed genes belonging to the “response to hypoxia”, “response to organic substance”, “response to protein stimulus”, the “transforming growth factor β receptor signaling pathway”, and “transmembrane receptor protein serine threonine kinase signaling pathway” ontology groups. The intensity of the edges reflects the strength of interaction score (Figure 5). It needs to be noted that *FOS, EGR1, ESR, SMAD4, AR, EGR2, INSR* and *STG2* show a number of functional links. These are not only defined interactions, but also those predicted to occur between the genes of interest. Another interesting aspect is the lack of interactions involving two of the analyzed genes: *CHRDL1* and *TACR3*. Finally, we investigated the functional interactions between chosen genes with REACTOME FIViz app to Cytoscape 3.6.0 software. The results were shown in Figure 6. The presented results bring attention to a number of functional interactions between the genes of interest. *FOS* activates/catalyzes *VEGFA, EGR2* and *SAMD4* expression. *SMAD4*, on the other hand, activates *ID1, ID2, AR* and *IHH*. *EGR1* further activates the expression of *MMP14, KLF10*, *VEGFA* and *AR*. Hence, it can be seen that *FOS*, *SMAD4*, *EGR1* and *VEGFA* exhibit the biggest network of functional links between the genes of interest.

**Figure 7:**
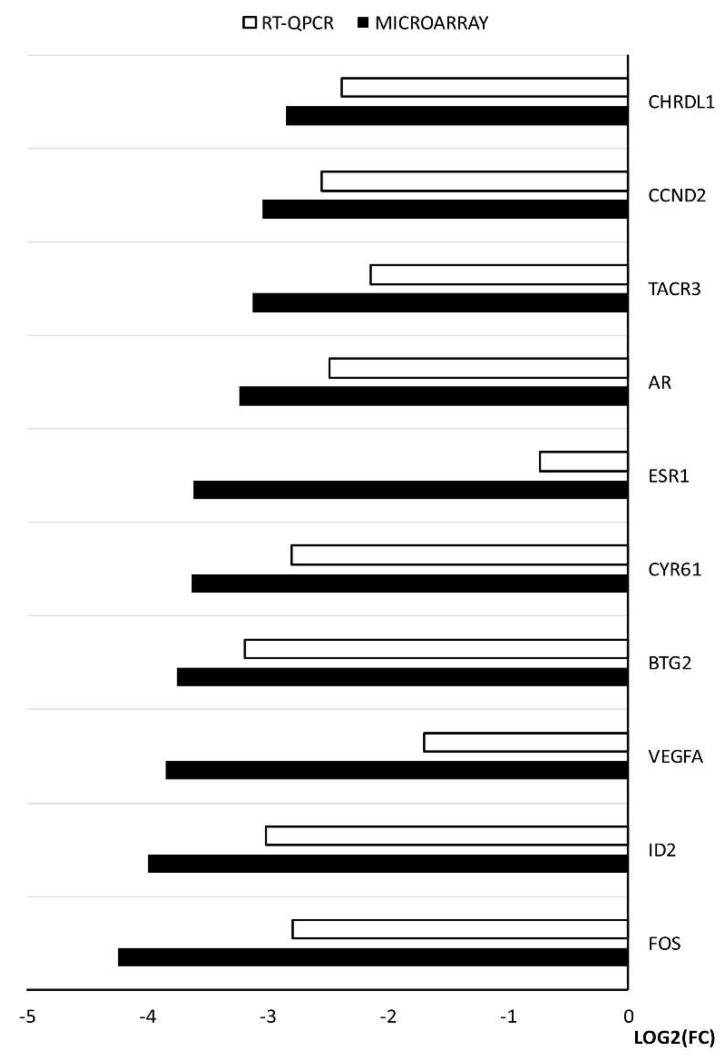
The results of RT-qPCR validation of the microarray analysis, presented in a form of a graph.

The changes in expression obtained from the microarray analysis were further validated using RT-qPCR. The results of the validation confirmed the direction of the changes in expression in all the cases. However, quantitative discrepancies were sometimes observed, mostly showing slightly lower values yielded from the RT-qPCR analysis. There were two genes that exhibited a larger difference between the two methods, *ESR1* and *VEGFA*. The results and comparison between the two methods are presented in the form of a graph (Figure 7).

**Figure 8:**
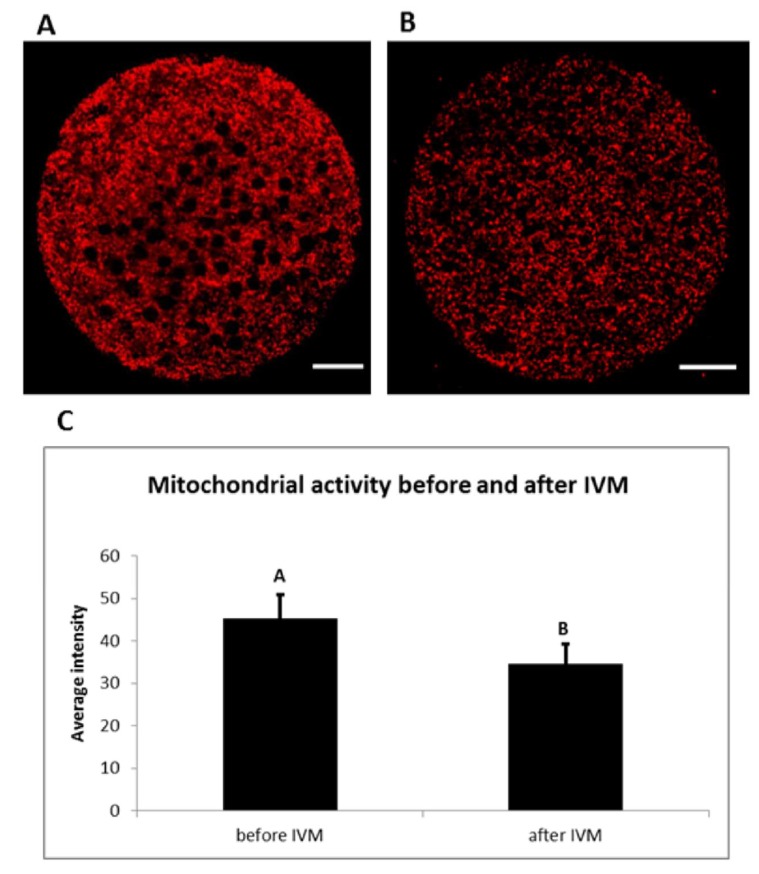
Mitochondrial activity during in vitro maturation (IVM). Representative images of porcine oocytes before (**A**) and after (**B**) in vitro maturation. Oocytes were stained by Mito Tracker Orange CMTM Ros (red color—active mitochondria). Scale bar represents 20 µm. The mean (±SD) of relative mitochondrial activity was significantly higher in oocytes before IVM than oocytes after IVM (**C**).

Mitochondrial activity of porcine oocytes changed during maturation (Figure 8). A significant decrease in mitochondrial activity was observed during in vitro maturation. This activity was expressed as an average intensity of fluorescent signal emitted when using dye specific for active mitochondria (Mito Tracker Orange CMTM Ros).

## 3. Discussion

In our research, we paid attention to the analysis of genes belonging to the “response to hypoxia”, “response to protein stimulus” and “response to organic substance” ontology groups, representing genes expressed in stress situations for which oocyte maturation and in vitro culture can be considered. The next two groups, “transmembrane protein receptor serine threonine kinase signaling pathway” and “transforming growth factor β receptor signaling pathway”, represent genes involved in pathways important for oocyte maturation. Analysis of these genes was carried out to assess the effect of their expression on protein response and mitochondrial activity status. Interdependences and interactions of the examined genes are presented in Figure 3, Figure 5 and Figure 6.

To describe and define new molecular markers expressed in oocytes and determine their ability to mature in in vitro conditions, in our studies, we performed the microarray analysis before and after in vitro maturation. By the examination of all 36 studied genes belonging to five ontology groups, after IVM we observed a down-regulation of expression. From that group, we decided to give further attention to the ten most changed genes: *FOS*, *ID2*, *VEGFA*, *BTG2*, *CYR61*, *ESR1*, *AR*, *TACR3*, *CCND2*, *CHRDL1*. The discussion will mainly focus on their functions in the processes occurring in mitochondria and the activity that they exhibit.

*FOS* (Fos proto-oncogene; AP-1 transcription factor subunit), belonging to all of the analyzed ontology groups except “response to hypoxia”, is recognized as a regulator of cell proliferation, transformation and differentiation. It has been proven that in some cases the expression of *FOS* can also be associated with programmed cell death through apoptosis [14]. Li et al. have shown that *FOS* mRNA is more stable in oocytes than in somatic cells, but the mechanism of this process has not yet been elucidated. They also established that the presence of maternal *FOS* mRNA in the oocyte is correlated with the expression of a protein encoded by the *MEX3C* gene [15]. A culture of blastocyst stage embryos in medium supplemented with PRDX II (Endogenous peroxiredoxin II) resulted in lower expression of *c-FOS*. In contrast, an increase in mitochondrial activity in response to expression of the Mitochondrial Transcription Factor A (*TFAM*) gene was observed [16]. These results indicate a decrease in the *FOS* gene expression and a simultaneous mitochondrial activity increase, which is in line with our results obtained at the stage of oocyte in vitro maturation supplemented with exogenous proteins. It also needs to be noted that *FOS* shows the biggest amount of functional interactions with the other genes of interest, promoting the expression of: *VEGFA*, *EGR2*, *SMAD4*. Next most downregulated gene, which belongs to the “response to protein stimulus” and “response to organic substance” ontology groups is *ID2* (inhibitor of DNA binding 2). This gene transcript promotes the expression of a protein that is a member of the DNA-binding inhibitor family. The expression of this helix-loop-helix protein is mainly induced by hypoxia, as well as other stresses [17] Increased *ID2* expression is also observed in ischemia, AMPK (5′ adenosine monophosphate-activated protein kinase) enzyme activation pathway, and in the induction of insulin pathway. The above features indicate the main role of *ID2* in metabolic cellular stresses [18]. In addition, the protein encoded by *ID2* is involved in the negative regulation of the cell differentiation process [19]. Our gene expression results are similar to those described by Budna et al. [20]. In the literature we can find information indicating that *ID2* is necessary to increase the number of cell receptors for the LH gonadotropin [21]. Under physiological conditions, loss of *ID2* occurs and is required in the process of trophoblastic stem cells (TSC) differentiation. *ID2* is one of the inhibitors of mitochondrial oxidative respiration. By modifying the mitochondrial function of the electron transport chain, it also regulates the reduced production of ATP in the cell. *ID2* decreases reactive oxygen species (ROS) production by lowering mitochondrial activity. Our results confirm a decrease in *ID2* gene expression during oocyte maturation. Developing oocytes have an increased demand for energy in the form of ATP. This is associated with mitochondrial activity increase and higher demand for oxygen. These results prove that oxygen deficiency is one of the limiting factors for in vitro culture. Interaction prediction analysis points at the activating role of *SMAD4* on the *ID2* gene. In turn, *SMAD4* is a central regulator of the main signaling pathway responsible for the achievement of developmental competence by the oocyte—TGFβ. The literature indicates that *SMAD4* deletion in granulosa cells disturbs the development of ovarian follicles. However, the exact role in the oocytes was not fully elucidated. [22]. The fall of *SMAD4* expression in the oocyte might be associated with transfer to the granulosa cell devoid environment. The research of Zhang et al. indicates that the silencing of *SMAD4* changes the expression of many genes involved in key biological processes associated with follicles [23]. The third gene, expression of which was the most down-regulated after oocyte maturation, is vascular endothelial growth factor A (*VEGFA)*. This gene is a member of the “response to hypoxia” ontology group, with an increase in its expression characteristic for cancer cells, in particular during the tumor progression process. *VEGFA* is a major factor promoting angiogenesis and endothelial cell migration during blood vessel formation [24,25]. VEGF is an important factor that inhibits the early stages of folliculogenesis [26]. There is evidence of the beneficial effect of *VEGF* gene expression on in vitro oocyte maturation efficiency, as well as further embryonic formation and development [27,28]. Wright GL et al. proved that, through the regulation of *AKT3* (AKT serine/threonine kinase 3), *VEGF* participates in the control of mitochondrial biological functions. Lowering *VEGF* expression may affect Akt3 silencing and lead to decreased expression of mitochondrial DNA genes [29]. These changes may have been caused by a reduced oxygen demand, which can also occur during oocyte in vitro maturation. As seen in the presented analyses, *VEGFA* is one of the genes of the highest decrease in expression. Furthermore, functional interaction analysis shows that *FOS* has an influence on the expression of this gene. The B cell translocation gene 2 (*BTG2*) participates in “response to organic substance” and is responsible for development, differentiation and cell death through apoptosis [30]. Expression of this gene is reduced in numerous human cancers. In the case of cancer cells, its decreased expression is associated with poor cell differentiation [31]. Another piece of research suggests that *BTG2* can cause cell apoptosis [32]. *BTG2* is one of the factors controlled by miR-663, involved in apoptotic permeabilization of mitochondrial outer membrane [33]. Therefore, the decreased expression of this gene in mature oocytes may be due to the fact that oocytes do not have the apoptotic cell characteristics and do not differentiate during maturation [34]. Literature data confirm the results obtained in the presented research. Additionally, it is predicted that this gene has a functional link to the androgen receptor (*AR*) gene (Figure 6). The next described gene involved in “response to protein stimulus” and to organic substance is cysteine rich angiogenic inducer 61 (*CYR61*). Protein encoded by this gene is expressed on the cell surface and mediates cell proliferation and adhesion. *CYR61* is identified as one of the promoters of cell migration in osteosarcoma [35]. Through the activation of *CASP3* (caspase-3), *Cyr61* can be a part of the mitochondrial membrane depolarization process. Compared to healthy cells, decreased expression of *Cyr61* is noted in cancerous tissues [36]. The next four identified genes, characteristic only for the “response to organic substance” ontology group, are estrogen receptor 1 (*ESR1*), *AR*, tachykinin receptor 3 (*TACR3*) and cyclin D2 (*CCND2*). *ESR1* encodes an estrogen receptor, with its main purpose being DNA binding and transcription activation. *ESR1* encodes receptor building proteins that, in combination with estrogen, creates the mechanism of cellular signaling process necessary for the development of reproductive functions. *ESR1* expression is observed in pre-granulosa cells and oocyte nuclei at early stages of fetal development [37]. In the case of the *ESR1* deletion, mitochondrial dysfunctions with increased ROS production were observed [38]. In this research, the expression of *ESR1* drops significantly after IVM. Previous literature reports note that this gene plays a role in oocyte maturation [39]. Activator of transcription, a protein encoded by the *AR* gene, is built from two domains: DNA-binding domain and androgen-binding domain. Female mice lacking *AR* expression in oocytes do not show reproductive dysfunctions [40]. Conditioned by the proapoptotic protein presence, cooperation of the *AR* gene with the RB (retinoblastoma) protein might lead to mitochondrial damage [41]. The *TACR3* gene encodes a neuropeptide receptor for the tachykinin neurokinin 3 (also known as neurokinin B). Its expression in oocytes before and after maturation is unexpected and may turn out to be a subject of further analysis. By binding to cyclin dependent kinase 4 (CDK4) or cyclin dependent kinase 6 (CDK6), the next analyzed gene, *CCND2*, builds a facilitating complex whose activity is required in the course of the cell cycle during the G1/S phase. It most prominently plays roles in proliferation of the reproductive cells. In addition, ovarian and testicular tumors express this gene at a high level. *CCND2* expression in human CCs during oocyte maturation indicates low competence of oocyte for further embryo development [42]. There is no clear evidence about the functions that this gene plays in matured oocytes. Its expression may result from close cooperation in two-way communication and signal exchange between oocyte and corona radiata cells. Last of the analyzed genes is chordin like 1 (*CHRDL1*). *CHRDL1* is a BMP4 (bone morphogenetic protein 4) antagonist, with its expression observed in response to hypoxia in the processes of retinal angiogenesis. Our results showed higher expression of this gene before the maturation process and confirm those presented by Budna J et al. [43]. *CHRDL1* has been shown to increase the proliferation of human mesenchymal stem cells, which may indicate its greater role in developing embryos rather than in oocyte maturation [44]. So far, no evidence that indicates its participation in processes related to the mitochondria functioning has been found. It needs to be noted that *TACR3* and *CHRDL1* do not show functional links to any of the selected genes.

Mitochondrial status is an important marker of cytoplasmic maturation of oocytes, as mitochondria are necessary for energy production, ATP concentration, apoptosis regulation and calcium homeostasis. Mitochondrial dysfunctions, including changes in the mitochondrial ultrastructure, an increase in mtDNA copy numbers, decreased ATP levels and tricarboxylic acid (TCA) cycle metabolites, correlate with reduced reproductive functions [45]. In our experiments, we have detected a decrease of mitochondrial activity after in vitro maturation. Similar results were presented in a previous study conducted on porcine BCB^+^ oocytes, showing high mitochondrial activity before in vitro maturation with a subsequent decrease after 22 h of this process [46]. This occurrence can be associated with the type of culture system, precise selection of oocytes before their using by BCB test and subsequent cultivation of cytoplasmatic mature porcine oocytes. Further analysis of these results can provide a stricter method of oocyte selection before IVF. However, to conduct functional analyses, oocytes obtained would need to be fertilised, followed by determination of expression change of the analyzed genes before and after embryonic genome activation. Hence, the study serves more as a basic molecular entry, needing further functional validation.

In conclusion, in our research we have attempted to identify new molecular markers of porcine oocyte maturity. Our analysis included the measurement of gene expression before maturation conducted in in vitro conditions and after its completion. All analyzed genes belonging to five functionally separated ontological groups: response to hypoxia (GO:0001666), “response to organic substance” (GO:0010033), “response to protein stimulus” (GO:0050896), “transforming growth factor β receptor signaling pathway” (GO:0007179) and “transmembrane receptor protein serine threonine kinase signaling pathway” (GO:0007178), showed decreased level of expression after in vitro oocytes. These genes were analyzed in the context of their expression induced by the culture conditions and its influence on the most important cell signaling pathways as well as the protein response and regulation of mitochondrial activity occurring in oocytes. Analysis of genes belonging to the selected by us ontological groups allows to state that mainly the *FOS*, *ID2*, *VEGFA* and *BTG2* genes can be considered as new markers of porcine oocyte maturity. This is evidenced by the lowest level of their transcripts in oocytes after IVM process as well as importance of these genes for oocytes and their mitochondria functioning. We suggest that understanding of expression of genes involved in biogenesis and activation of the mature oocytes’ mitochondria may be crucial for comprehending their potential for fertilization. Our research may be helpful in the subsequent establishment of mitochondrial molecular markers of metaphase II oocytes. However, the presented study needs to be considered as a basic molecular entry and needs further validation to confirm that the results obtained translate into in vivo and clinical situations.

## 4. Material and Methods

### 4.1. Experimental Design

Oocytes were collected and subjected to two Brilliant Cresyl Blue (BCB) tests and divided into two groups. The first group (“before IVM”) included oocytes graded as BCB-positive (BCB^+^) and directly exposed to microarray assay and RT-qPCR. The second group (“after IVM”) included BCB^+^ oocytes which were then in vitro matured and, if classified as BCB^+^ in second BCB test, passed to molecular analyses.

### 4.2. Animals

A total of 45 pubertal crossbred Landrace gilts bred on a commercial local farm were used in this study. They had a mean age of 155 days (range 140–170 days) and a mean weight of 100 kg (95–120 kg). All animals were bred under the same conditions and fed the same forage (depending on age and reproductive status). Experiments were approved by the Poznan University of Medical Sciences Bioethical Committee (Resolution No. 32/2012, approved on 1/6/2012).

### 4.3. Collection of Porcine Ovaries and Cumulus-Oocyte-Complexes (COCs)

The ovaries and reproductive tracts were recovered at slaughter and transported to the laboratory within 40 min at 38 °C in 0.9% NaCl. To provide optimal conditions for subsequent oocyte maturation and fertilization in vitro, the ovaries of each animal were placed in a 5% fetal bovine serum solution (FBS; Sigma-Aldrich Co., St. Louis, MO, USA) in PBS. Single large follicles (>5 mm) were opened by puncturing with a 5 mL syringe and 20-G needle in a sterile Petri dish, and COCs were recovered. The COCs were washed three times in modified PBS supplemented with 36 µg/mL pyruvate, 50 µg/mL gentamycin, and 0.5 mg/mL BSA (Sigma-Aldrich). The COCs were selected under an inverted microscope (Zeiss, Axiovert 35; Lübeck, Germany), counted, and morphologically evaluated. Only COCs of grade I, possessing homogeneous ooplasm and uniform, compact cumulus cells were considered for further use, resulting in a total of 300 grade I oocytes (3 × *n* = 50 “before IVM” group, 3 × *n* = 50 “after IVM” group).

### 4.4. Assessment of Oocyte Developmental Competence by BCB Test

A Brilliant Cresyl Blue (BCB) test was used for the assessment of porcine oocytes’ quality and maturity [47]. The glucose-6-phosphate (G6PDH) enzyme converts BCB stain from blue to colorless. In oocytes that completed the growth, activity of the enzyme decreases and the stain cannot be reduced, resulting in blue oocytes (BCB^+^). To perform the BCB staining test, oocytes were washed twice in modified Dulbecco’s Phosphate Buffered Saline (DPBS) commercially supplemented with 0.9 mM calcium, 0.49 mM magnesium, 0.33 mM pyruvate, and 5.5 mM glucose (Sigma-Aldrich), and additionally with 50 IU/mL penicillin, 50 µg/mL streptomycin (Sigma-Aldrich), and 0.4% Bovine Serum Albumin (BSA) (*w/v*) (Sigma-Aldrich). They were then treated with 13 µM BCB (Sigma-Aldrich) diluted in DPBS at 38.5 °C, 5% CO_2_ for 90 min. After treatment, the oocytes were transferred to DPBS and washed twice. During washing, the oocytes were examined under an inverted microscope and classified as stained blue (BCB^+^) or colorless (BCB^–^). Only the granulosa cell-free BCB^+^ oocytes were used for subsequent molecular analyses (“before IVM” group) or IVM followed by second BCB test and molecular analyses (“after IVM” group). After the first BCB test, 300 BCB^+^ oocytes were selected for the study. One hundred and fifty were directly analyzed as the “before IVM” group, while 150 were passed to subsequent maturation. After maturation, around 70% (105) oocytes were determined as BCB^+^ and subjected to molecular analyses as the “after IVM” group.

### 4.5. In Vitro Maturation of Porcine Cumulus-Oocyte-Complexes (COCs)

After the first BCB test, the BCB^+^ COCs were subjected to IVM. The COCs were cultured in Nunclon™Δ 4-well dishes (Thermo Fisher Scientific, Waltham, MA, USA) in 500 μL standard porcine IVM culture medium: TCM-199 (tissue culture medium) with Earle’s salts and l-glutamine (Gibco BRL Life Technologies, Grand Island, NY, USA), supplemented with 2.2 mg/mL sodium bicarbonate (Nacalai Tesque, Inc., Kyoto, Japan), 0.1 mg/mL sodium pyruvate (Sigma-Aldrich, St. Louis, MO, USA), 10 mg/mL BSA (Bovine Serum Albumin) (Sigma-Aldrich, St. Louis, MO, USA), 0.1 mg/mL cysteine (Sigma-Aldrich, St. Louis, MO, USA), 10% (*v*/*v*) filtered porcine follicular fluid, and gonadotropin supplements at final concentrations of 2.5 IU/mL hCG (human Chorionic Gonadotropin) (Ayerst Laboratories, Inc., Philadelphia, PA, USA) and 2.5 IU/mL eCG (equine Chorionic Gonadotropin) (Intervet, Whitby, ON, Canada). Wells were covered with mineral oil overlay and cultured at 38 °C under 5% CO_2_ in air for 22 h, and then for additional 22 h in medium without hormones. After cultivation, the second BCB staining test was performed, and BCB^+^ oocytes were used for further molecular analyses. Before RNA extraction COCs were incubated with bovine testicular hyaluronidase (Sigma-Aldrich) for 2 min at 38 °C to separate cumulus and granulosa cells. The cumulus cells were removed by mechanical displacement by means of a small diameter glass micropipette.

### 4.6. RNA Extraction from Porcine Oocytes

Total RNA was extracted from samples using the TRI Reagent (Sigma-Aldrich), and RNeasy MinElute cleanup Kit (Qiagen, Hilden, Germany). The amount of total mRNA was determined using optical density at 260 nm, and the RNA purity was estimated using the 260 nm/280 nm absorption ratio (higher than 1.8) (NanoDrop spectrophotometer, Thermo Scientific, ALAB, Warsaw, Poland). The RNA integrity and quality were checked on a Bioanalyzer 2100 (Agilent Technologies, Inc., Santa Clara, CA, USA). The resulting RNA integrity numbers (RINs) were between 8.5 and 10 with an average of 9.2 (Agilent Technologies, Inc.). The RNA in each sample was diluted to a concentration of 100 ng/μL with an OD260/OD280 ratio of 1.8/2.0. From each RNA sample, 100 ng of RNA was taken for the further molecular analysis.

### 4.7. Microarray Expression Analysis and Statistics

Experiments were performed in three replicates as described in our previous works [48,49,50,51,52]. Total RNA (100 ng) from each pooled sample was subjected to two round sense cDNA amplification (Ambion^®^ WT Expression Kit). The obtained cDNA was used for biotin labeling and fragmentation through Affymetrix GeneChip^®^ WT Terminal Labeling and Hybridization (Affymetrix, Santa Clara, CA, USA). Biotin-labeled fragments of cDNA (5.5 μg) were hybridized to Affymetrix^®^ Porcine Gene 1.1 ST Array Strip (48 °C/20 h). Then, the microarrays were washed and stained according to the technical protocol, using Affymetrix GeneAtlas Fluidics Station. The array strips were scanned employing the Imaging Station of the GeneAtlas System. The preliminary analysis of the scanned chips was performed using the Affymetrix GeneAtlas™ Operating Software. Quality of gene expression data was checked according to quality control criteria provided by the software. The obtained CEL files were imported into downstream data analysis software.

All analyzes were performed using the BioConductor software, based on the statistical R programming language. For background correction, normalization and summation of raw data, the Robust Multiarray Averaging (RMA) algorithm implemented in “affy” package of BioConductor was applied. Biological annotation was taken from the BioConductor “oligo” package, where the annotated data frame object was merged with a normalized data set, leading to a complete gene data table. Statistical significance of the analyzed genes was performed using moderated t-statistics from the empirical Bayes method. Obtained *p*-value was corrected for multiple comparisons using the Benjamini and Hochberg’s false discovery rate. The selection of significantly changed gene expression was based on *p* value beneath 0.05 and expression fold higher than |2|.

Functional annotation clustering of differentially expressed genes was performed using DAVID (Database for Annotation, Visualization and Integrated Discovery). Gene symbols for down-regulated genes from each of the compared groups were loaded to DAVID by “RDAVIDWebService” BioConductor package. For further analysis we have chosen the enriched GO terms which contained at least 5 genes and exhibited a *p*-value (Benjamini) lower than 0.05. The enriched GO terms were subjected to a hierarchical clusterization algorithm and presented as heatmaps.

Subsequently we analyzed the relation between the genes belonging to chosen GO terms using the GOplot package [53]. The package calculated the z-score: the number of up-regulated genes minus the number of down-regulated genes divided by the square root of the count. This information allowed for estimation of the directions of changes of each gene-ontology term.

Among the genes that build the chosen GO terms, we have chosen the 10 that were the most down-regulated. Interactions between chosen differentially expressed genes/proteins belonging to ontology group were investigated using the STRING10 software (Search Tool for the Retrieval of Interacting Genes; STRING Consortium, Lausanne, Switzerland). A list of gene names was used as query for an interaction prediction. The search criteria were based on co-occurrences of genes/proteins in scientific texts (text mining), co-expression and experimentally observed interactions. The results of this analysis generated a gene/protein interaction network where the intensity of the edges reflected the strength of the interaction score. Besides interaction prediction, STRING also allowed us to perform functional enrichments of GO terms based on previously uploaded gene sets.

Finally, the functional interactions between genes that belong to the chosen GO BP terms were investigated by REACTOME FIViz application to the Cytoscape 3.6.0 software (The Cytoscape Consortium, San Diego, CA, USA). The ReactomeFIViz app is designed to find pathways and network patterns related to cancer and other types of diseases. This app accesses the pathways stored in the Reactome database, allowing to perform a pathway enrichment analysis for a set of genes, visualize hit pathways using manually laid-out pathway diagrams directly in Cytoscape, and investigate functional relationships among genes in hit pathways. The app can also access the Reactome Functional Interaction (FI) network, a highly reliable, manually curated pathway-based protein functional interaction network covering over 60% of human proteins.

### 4.8. Real-Time Quantitative Polymerase Chain Reaction (RT-qPCR) Analysis

Total RNA was isolated from oocytes before and/or after IVC. The RNA samples were re-suspended in 20 µL of RNase-free water and stored in liquid nitrogen. The samples were treated with DNase I and reverse-transcribed (RT) into cDNA. RQ-PCR was conducted in a LightCycler real-time PCR detection system (Roche Diagnostics GmbH, Mannheim, Germany) using SYBR^®^ Green I as a detection dye, with target cDNA quantified using the relative quantification method. The relative abundance of *FOS*, *ID2*, *VEGFA*, *BTG2*, *CYR61*, *ESR1*, *AR*, *TACR3*, *CCND2*, *CHRDL1* transcripts in each sample was standardized to the internal standard of glyceraldehyde-3-phosphate dehydrogenase (GAPDH). For amplification, 2 µL of cDNA solution was added to 18 µL of QuantiTect^®^ SYBR^®^ Green PCR (Master Mix Qiagen GmbH) and primers (Table 2). One RNA sample of each preparation was processed without the RT-reaction to provide a negative control for subsequent PCR.

To quantify specific genes in the oocyte, expression levels of specific oocyte mRNAs were calculated relative to PBGD and ACTB. To ensure the integrity of these results, the additional housekeeping gene 18S rRNA was used as an internal standard to demonstrate that PBGD and ACTB mRNAs were not differentially regulated in the groups of oocytes. The 18S rRNA expression was identified as an appropriate housekeeping gene for the use in quantitative PCR studies. Expression of PBGD, ACTB, and 18S rRNA mRNAs was measured in samples from isolated oocytes.

### 4.9. Oocyte Mitochondrial Activity Examination

**Figure 9:**
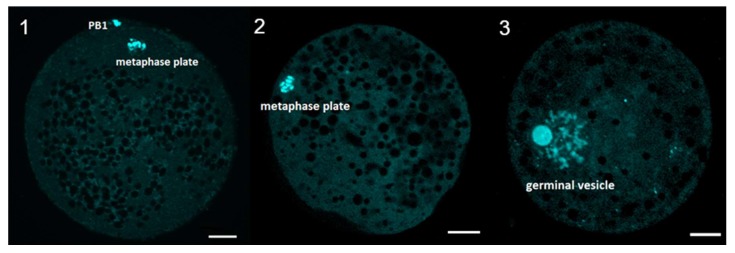
Evaluation of meiotic maturation after IVM. Representative images of porcine oocyte after in vitro maturation. (**1**) mature oocyte at metaphase of second meiotic division (**2**) immature oocyte at metaphase of first meiotic division (**3**) immature oocyte at GV stage (germinal vesicle). Oocytes were stained by DAPI. Scale bar represents 20 µm. PB1—first polar body.

The selection of oocytes for this experiment was conducted in the way of DAPI staining, with characteristics described on the figure below (Figure 9) serving as indicators of particular maturational stages. Around 85% of the total “after IVM” oocytes were proven to be fully mature and were subsequently used to assess the mitochondrial activity of the mature oocytes in the later stages of the experiment.

Oocytes in the germinal vesicle stage and mature oocytes were denuded of cumulus cells manually in IVM culture medium with 0.1% (*w*/*v*) hyaluronidase (Sigma Aldrich). The active mitochondria of the oocytes were stained in PBS supplemented with 0.4% BSA and 200 nM MitoTracker Orange CMTM Ros dye (Molecular Probes, Eugene, OR, USA) for 30 min at 38 °C. Only respiring mitochondria were stained with this cell-permeant mitochondrial-specific dye. After washing, the oocytes were fixed in 3.7% paraformaldehyde for 60 min at room temperature. They were washed in PBS and mounted on glass slides, avoiding oocyte compression, using Vectashield medium (Vector Lab, Burlingame, CA, USA) containing 1 μM of DNA dye (SYTOX Green, Invitrogen; Carlsbad, CA, USA) specific for dyeing of chromatin. The slides were stored below 0 °C until examined. The oocytes were examined with the use of a laser scanning confocal microscope (Leica TCS SP2 AOBS; Leica, Heidelberg, Germany) equipped with Ar and HeNe lasers. The 488 nm excitation band and 570–667 nm detector were used for lipid droplets visualization and 633 nm excitation band and 635–713 nm detector for detection of chromatin. The 40× Leica HCX PL APO CS objective, pinhole, offsets, gain and AOBS were adapted. These parameters were kept throughout the whole experiment. The oocytes were scanned in equatorial optical section, micro photographs were saved and processed using the NIS–Elements AR 3.00 software (Nikon, Melville, NY, USA). Mean intensity of signal from sections was analyzed. The data was analyzed with Fisher’s least significant difference (LSD) test using ANOVA SPSS version 11.5 for Windows (SPSS, Inc., Chicago, IL, USA). Differences at *p* < 0.05 were considered statistically significant.

## Figures and Tables

**Table 1:** Gene symbols, fold changes in expression, corrected *p* values and LogFC of studied genes.

Official Gene Symbol	Fold Change	Adjusted *p* Value	logFC
*FOS*	0.052794356	4.74 × 10^−5^	−4.243472475
*ID2*	0.062979704	4.74 × 10^−5^	−3.988969222
*VEGFA*	0.069689389	0.001912689	−3.84291719
*BTG2*	0.074386393	9.55 × 10^−5^	−3.748817455
*CYR61*	0.080657036	7.54 × 10^−5^	−3.632055792
*ESR1*	0.081629841	0.000522187	−3.614759536
*AR*	0.1059863	0.000138367	−3.238050297
*TACR3*	0.115060322	0.000148036	−3.119537684
*CCND2*	0.121809064	0.000178804	−3.0373066
*CHRDL1*	0.139364543	4.74 × 10^−5^	−2.843064537
*EGR2*	0.165503832	0.007949861	−2.595063475
*EDNRA*	0.166939028	0.00185422	−2.582606817
*ANGPTL4*	0.183631311	0.000513422	−2.44511602
*TGFBR3*	0.196522244	0.000405979	−2.347235474
*FST*	0.224696558	0.000364693	−2.153950068
*MCL1*	0.244179957	0.001775249	−2.033983311
*IHH*	0.304995843	0.000551261	−1.713138513
*INSR*	0.31601561	0.001912689	−1.661932271
*ZCCHC11*	0.3216223	0.019809962	−1.636560654
*ID1*	0.335473139	0.003974331	−1.575730839
*TXNIP*	0.355538611	0.000780875	−1.491921851
*SMAD4*	0.367802201	0.001238681	−1.442997981
*MAP3K1*	0.36876538	0.024748462	−1.439224873
*EGR1*	0.376128185	0.005477006	−1.410703676
*PDLIM1*	0.380689412	0.001429255	−1.393313651
*UBE2B*	0.382779667	0.041104659	−1.385413899
*PHIP*	0.385682339	0.02111605	−1.374515012
*ECE1*	0.395387731	0.001177804	−1.338659992
*IGFBP7*	0.403759522	0.002496043	−1.30843181
*KLF10*	0.405438718	0.00684513	−1.302444226
*EIF2AK3*	0.41888965	0.008422055	−1.255357857
*VCP*	0.435612412	0.007402292	−1.198883032
*HSPA4*	0.441182204	0.002321468	−1.180553494
*SERPINH1*	0.467321273	0.006338248	−1.097513384
*PLD1*	0.468341554	0.011044722	−1.094367046
*MMP14*	0.488721147	0.038060423	−1.032916564

**Table 2:** Sequences of primers used for RT-qPCR validation of the microarray results.

Gene	Gene ID	Primer Sequence (5′–3′)	Product Size (bp)
*FOS*	100144486	AGAATCCGAAGGGAAAGGAACTTCTCCTTCAGCAGGTTGG	150
*ID2*	654298	CCAGTGAGGTCCGTTAGGAAGACAATAGTGGGGTGCGAGT	243
*VEGFA*	397157	CTACCTCCACCATGCCAAGTACACTCCAGACCTTCGTCGT	232
*BTG2*	100048932	TGGTTTCCTGAAAAGCCATCGGACACTTCATAGGGGTCCA	150
*CYR61*	100153791	GAGCCTCGCGTTCTCTACACTGCATCTCTTGCCCTTTTTC	217
*ESR1*	397435	CGTCCAAGCTCAAAGAGACCCGAAGAATGTGCTCGATGAA	160
*AR*	397582	GAACCTACCAGGGACCATGTCTGTTTCCCTTCAGCAGCTC	156
*TACR3*	100521983	GGTCCCAAACAACACTTCACTGCCTTTAGCTGCTCATGGTA	161
*CCND2*	397162	GGCAAGTTGAAGTGGAACCTTGGCGAACTTGAAGTCAGTG	154
*CHRDL1*	100521058	TTCCTAGAAGGAAGCAAGACAGCGTTCTCTGAGCAGATGCAG	151
